# Supplementary material for: Serum Cytokines During Acute Respiratory Infection and Relationship to Age
Source: J Cell Immunol. Author manuscript; Available in PMC 2026 Jan 17. (PMC12811030; doi:10.33696/immunology.7.234)
Supplement: JCI-25-234-Supplementary_File [file NIHMS2132277-supplement-JCI-25-234-Supplementary_File.pdf]

Gupta A, Maecker HT. Serum Cytokines During Acute Respiratory Infection and Relationship to Age. J Cell Immunol. 2025;7(4):139–145.

Supplemental Tables

Supplementary Table 1. List of all biomarkers with significant difference among distribution for healthy and infected cohorts.

Biomarker	P_Value (adjusted)
IP-10	1.26E-11
RANTES/CCL5	0.000283
IL1RA	0.00407
IL7	0.00451
GROA/KC/CXCL1	0.00645
HGF	0.01729
ICAM1	0.01058
IFNA	0.04976
IL31	0.00921
TNFA	0.01121
VCAM1	0.01219

Supplemental Table 2. Cytokines with significant slopes among the healthy subjects.

Biomarker	P-Value (adjusted)	P-Value (unadjusted)	Slope for Healthy Subjects
EOTAXIN/CCL11	2.29E-09	5.72E-10	2.788
LEPTIN	1.78E-07	4.44E-08	37.974
MCP1/CCL2	1.19E-07	5.94E-08	4.445
VCAM1	1.45E-06	7.27E-07	14.356
BDNF	1.91E-06	9.53E-07	28.953
CD40L	p > 0.05	0.044015	3.4463
CSF2/GMCSF	3.94E-05	1.54E-05	41.704
HGF	0.000347	0.000173	1.3406
ICAM1	0.00543	0.002717	11.34
IL17F	p > 0.05	0.035854	2.0015
IL22	0.01417	0.007084	1.0307
IL9	p > 0.05	0.049673	0.3142

IP-10/CXCL10	0.000153	3.82E-05	2.4805
MIG/CXCL9	0.000822	0.000206	2.3721
MIP1A/CCL3	p > 0.05	0.01517	3.4432
PAI1/SERPINE1	1.07E-05	5.34E-06	16.945
PDGFBB	1.87E-06	9.33E-07	2.3926
PIGF1	p > 0.05	0.03575	1.1613
RANTES/CCL5	1.65E-05	8.25E-06	7.5756
RESISTIN	0.00533	0.002665	10.552
VEGF	0.02774	0.013871	3.4894
